# Influence of Polymer Charge on the Localization and Dark- and Photo-Induced Toxicity of a Potential Type I Photosensitizer in Cancer Cell Models

**DOI:** 10.3390/molecules25051127

**Published:** 2020-03-03

**Authors:** Mikael Lindgren, Odrun A. Gederaas, Monica Siksjø, Tom A. Hansen, Lena Chen, Bastien Mettra, Chantal Andraud, Cyrille Monnereau

**Affiliations:** 1 Department of Physics, Faculty of Natural Sciences, Norwegian University of Science and Technology, Gløshaugen, NO-7491 Trondheim, Norway; odrun.gederaas@ntnu.no (O.A.G.); m.siksjo@gmail.com (M.S.); tomhans@viken.no (T.A.H.); 2 Department of Clinical and Molecular Medicine, Faculty of Medicine and Health Sciences, Norwegian University of Science and Technology, Laboratoriesentret 5, NO-7491 Trondheim, Norway; 3 Laboratoire de Chimie, CNRS UMR 5182, ENS de Lyon, Université Lyon 1, F-69342 Lyon, France; chenlena93@gmail.com (L.C.); bastien.mettra@univ-lyon1.fr (B.M.); chantal.andraud@ens-lyon.fr (C.A.); cyrille.monnereau@ens-lyon.fr (C.M.)

**Keywords:** photo-dynamic therapy, anthracene, singlet oxygen luminescence, CHO-K1 cells, cell localization

## Abstract

A current trend within photo-dynamic therapy (PDT) is the development of molecular systems targeting hypoxic tumors. Thus, type I PDT sensitizers could here overcome traditional type II molecular systems that rely on the photo-initiated production of toxic singlet oxygen. Here, we investigate the cell localization properties and toxicity of two polymeric anthracene-based fluorescent probes (neutral Ant-PHEA and cationic Ant-PIm). The cell death and DNA damage of Chinese hamster ovary cancer cells (CHO-K1) were characterized as combining PDT, cell survival studies (MTT-assay), and comet assay. Confocal microscopy was utilized on samples incubated together with either DRAQ5, Lyso Tracker Red, or Mito Tracker Deep Red in order to map the localization of the sensitizer into the nucleus and other cell compartments. While Ant-PHEA did not cause significant damage to the cell, Ant-PIm showed increased cell death upon illumination, at the cost of a significant dark toxicity. Both anthracene chromophores localized in cell compartments of the cytosol. Ant-PIm showed a markedly improved selectivity toward lysosomes and mitochondria, two important biological compartments for the cell’s survival. None of the two anthracene chromophores showed singlet oxygen formation upon excitation in solvents such as deuterium oxide or methanol. Conclusively, the significant photo-induced cell death that could be observed with Ant-PIm suggests a possible type I PDT mechanism rather than the usual type II mechanism.

## 1. Introduction

Photo-dynamic therapy (PDT) is a collective term for the process of using light in oxidative photo-chemical reactions to alter the biological functions of a living system. The phenomenon is currently used as an alternative approach in the treatment of a number of diseases. Usually, some photosensitizer (PS) is added to target and/or accelerate the process and to stimulate, e.g., cancer cell death [1]. The overall outcome is both dependent on the localization of the PS in organelles such as mitochondria, lysosomes, internal cell membranes, or other sub-cellar compartments, as well as of the intrinsic cytotoxicity of the chromophore. The latter generally depends on the propensity to generate reactive oxygen species upon illumination (type II PDT) although alternative photo-redox mechanisms exist (type I) [2]. Interestingly, while most PDT probes developed either in the context of fundamental research and clinical applications focus on a type II PDT mechanism (i.e., energy transfer to triplet molecular oxygen, resulting in the build-up of cytotoxic ^1^O_2_ within the cell), tumor micro-environments are often hypoxic, which may limit the success of this therapeutic approach, especially in the case of advanced cancers [3]. Thus, the development of complementary strategies relying on type I PDT (direct redox mechanism between the excited state of the PS and its environment such as water or biomolecules) is a valuable alternative to circumvent this limitation [4,5]. After or during PDT, cells undergo apoptosis or necrosis. Apoptosis is a rapidly programmed cell death, generally believed to be related to base oxidative damage, strand breaks or cross-links [6,7]. The nucleus will show a characteristic degradation onto globules and internucleosomal units [8]. Necrosis on the other hand, is accidental, and often follows massive tissue injury (such as mechanical trauma or exposure to a toxic agent). The damage is too severe for the cell to maintain its level of normal activity and falls apart [9].

One of the most stringent prerequisites for an optimized photosensitizer in the context of PDT is that it undergoes fast and efficient accumulation into its target biological compartment. In the case of a tumor, the penetration efficiency of the PS inside the cancer cell and its subsequent localization within the cell compartments are two crucial issues [10]. We recently reported on a simple yet efficient strategy, based on the controlled introduction of water-soluble polymer chains, allowing for the bio compatibilization of a variety of two-photon fluorophores and photosensitizers at a moderate synthetic cost [11,12,13]. However, it was shown that the resulting objects did neither display particularly efficient accumulation within cancer cells, nor specificity to a given cellular compartment. Indeed, a rather homogeneous staining of the cytoplasm was observed. It is well established that the introduction of cationic moieties onto chromophore molecules favors their diffusion through cell membranes [14,15] and their accumulation within relevant organelles, such as mitochondria [16,17] or nuclei [18,19]. We therefore tested the influence of the substitution of hydroxyl groups by imidazolium moieties along the polymer chain, following a recently reported strategy [20,21,22] on the targeting abilities of the chromophore.

**Figure 1:**
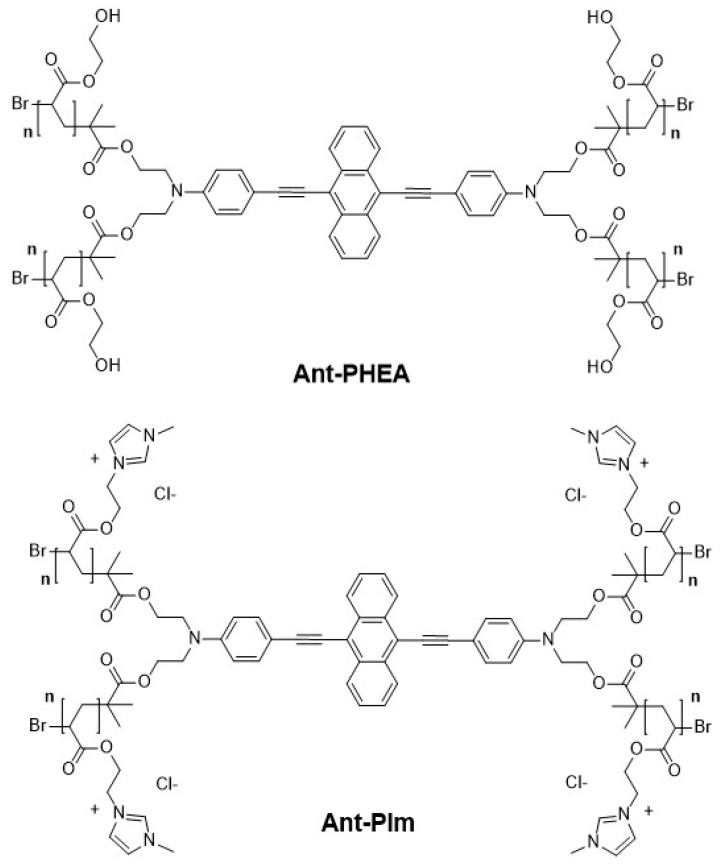
Hydroxyl-substituted (Ant-PHEA) and the imidazolium-substituted (Ant-PIm) anthracene chromophore.

In this paper, we report on a comparative study on the localization of the polycationic imidazolium-substituted probe (Ant-PIm) vs. the hydroxyl-substituted probe (Ant-PHEA) with structures shown in Figure 1, and on their respective dark and light-induced cytotoxicity’s in CHO-K1 cells. The anthracene motif was chosen as it has long been well established that its excited state photophysics involve a long lived triplet state that can diffuse over long distances in oxygen-deprived environments, a property that currently finds promising applications in triplet–triplet annihilation processes [23] and might, in our opinion, bring significant advantage for type I PDT. To this end, we developed an algorithm that facilitates the objective calculation of the colocalization factors of the studied probe, in comparison with commercial specific fluorescent organelles labels. We establish that Ant-PIm presents significant benefits regarding both issues, which confirms the interest of the introduction of cationic groups along the polymer chain for the design of future photosensitizers relying on a similar approach, and shed some light on the origin of the photoinduced toxicity, for which we bring compelling evidence that it does not rely on the production of singlet oxygen. This may suggest a possible type I mechanism as the origin of the PDT effect.

## 2. Results

### 2.1. Spectroscopy

In order to assess the basic photo-physical properties relevant for PDT and fluorescence imaging, absorption and emission spectra, along with direct formation of singlet oxygen from energy transfer from the PS to molecular oxygen, were investigated. 

**Figure 2:**
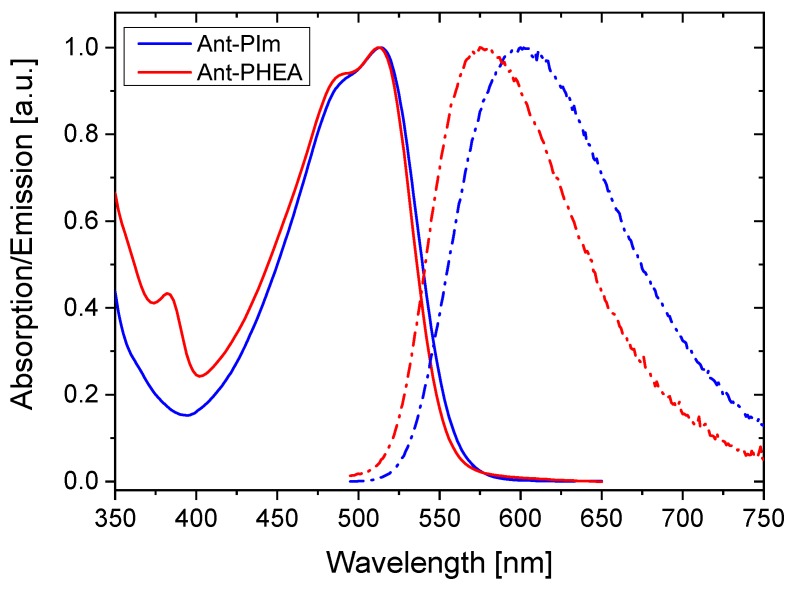
Normalized absorption (solid lines) and emission (dashed-dots) spectra of a diluted solution of Ant-PIm (blue) and Ant-PHEA (red) in physiological serum. The concentration in all experiments is ca 10^−5^ M.

The lower energy absorption bands of Ant-PIm and Ant-PHEA in water are very similar, as shown in Figure 2. In each case, the spectrum is dominated by a broad, structureless band, centered at 510 nm, in agreement with the intramolecular charge transfer nature of the associated electronic transition [24]. A sub-structure can be discerned and is slightly more pronounced for Ant-PHEA as compared to Ant-PIm but gives rise in both cases to a shoulder in the blue edge of the band (470 nm). The small 375 nm centered transition, visible in the spectrum of Ant-PHEA, and attributed in previous papers to a forbidden (centrosymmetry) S0→S2 transition vanishes in Ant-PIm, indicating slight changes in the conformational freedom of the molecule. Molar extinction coefficients are, within experimental errors, similar in both cases (approx. 5 × 10^4^ M^−1^ cm^−1^). In contrast, the position of the fluorescence band is red-shifted by almost 15 nm between Ant-PHEA and Ant-PIm, under similar solvent and temperature conditions, again witnessing slight differences in the polarity environment, which may arise from the substitution of the OH moieties of PHEA by cationic imidazolium groups. Within the experimental error, the quantum efficiency in water was the same for both chromophores (Ø = 0.3; using fluorescein as reference).

**Figure 3:**
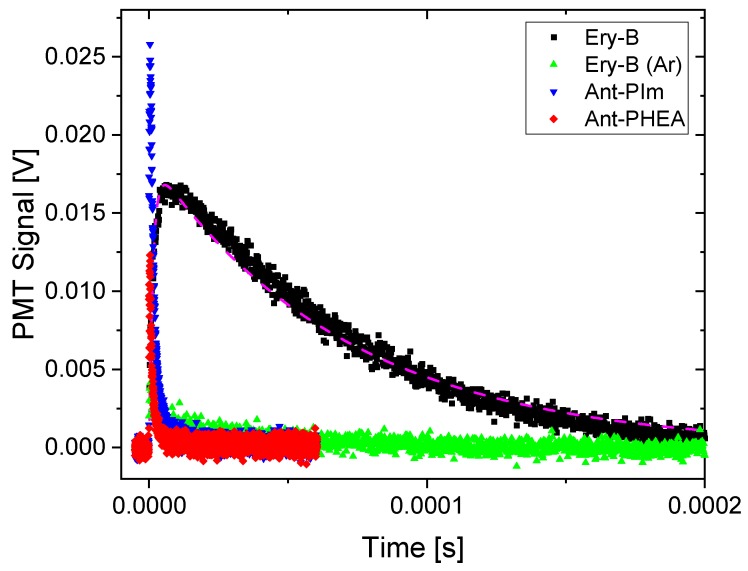
Transient luminescence at 1275 nm recorded for Ant-PIm (blue), Ant-PHEA (red), and Erythrosin B (black) in D_2_O. The weak signal obtained for Ar-purged D_2_O/Erythrosin B is also shown (green). The dashed curve is a simulation with parameters, as described in the text.

Singlet oxygen formation was measured from the direct monitoring of its transient luminescence at 1275 nm against the well-known reference compound Erythrosin B [25]. Such transients are shown in Figure 3. 

Erythrosin shows a typical signal characterized by a sharp rise time, reflecting the quenching of the initiating triplet state, and a slower decay, reflecting the lifetime of the singlet oxygen in the solvent. The transient decay shape is usually not very dependent on the solute photosensitizer, as it mostly depends on the natural lifetime of singlet oxygen and its interaction with the solvent [26,27]. 

In the simulation of the Erythrosin transient shown as the dashed curve in Figure 3, 1.8 μs was used for the triplet decay (i.e., the apparent rise-time) and 72 μs for the decay of the singlet oxygen luminescence (the long tail progressing with time). The transient luminescence at 1275 nm for Ant-PIm and Ant-PHEA is dramatically different, with very short-lived mono-decay components being 1.6 and 1.0 μs, respectively. This quicker transient did not change upon purging the solvent with Argon, in contrast to the transient of Erythrosin B that almost completely disappeared (green dots in Figure 3). To see if there was a solvent effect, the same experiments were repeated using methanol as a solvent (plots not shown). Here, both the singlet oxygen decay and triplet decays of Erythrosin B were found to be shorter, 17 μs and 0.3 μs, respectively, whereas the transients of Ant-PIm remained the same and the transient of Ant-PHEA increased its time-constant to 2.4 μs. Conclusively, no characteristic singlet oxygen luminescence was observed for Ant-PIm or Ant-PHEA in D_2_O or methanol, the latter two being common solvents for singlet oxygen detection.

### 2.2. Toxicity and Phototoxicity

**Figure 4:**
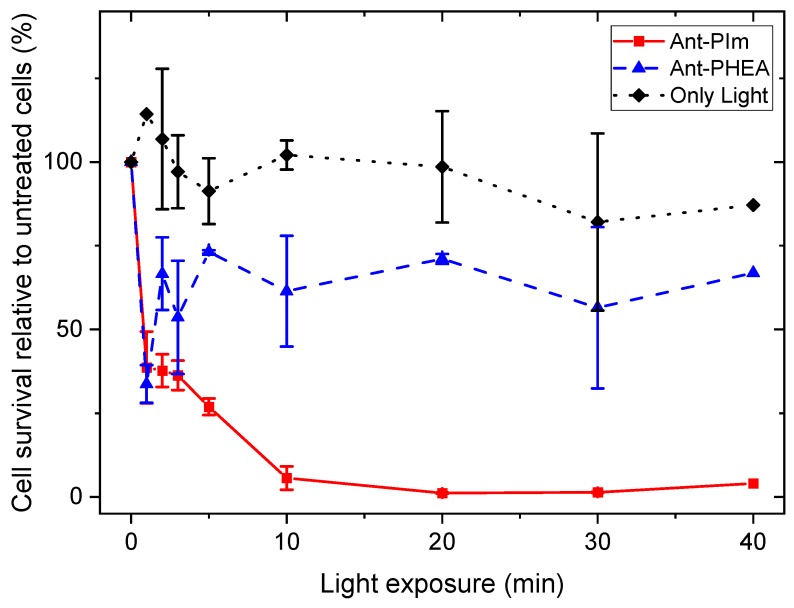
Effects of photodynamic therapy (PDT) using the anthracene derivatives Ant-PIm (10 µM, 24 h) and Ant-PHEA (10 µM, 24 h) on the cell survival of CHO-K1 hamster ovary cells, as a function of blue light exposure (0–30 min), as described in “Material and Methods”. The cell viability was measured by MTT assay 24 h post light exposure and was normalized to 100% viability for untreated cells incubated in a drug-free medium. The results were present as mean values ± SD from three separate experiments.

To investigate the photosensitizing effects of the chromophores, CHO-K1 cells were treated with Ant-PIm and Ant-PHEA (10 μM, 24 h) and exposed to blue light (0–40 min, 13 mW/cm^2^), the wavelength of which matched the spectra shown in Figure 2. The cell viability, as a % of untreated CHO-K1 cells, is presented in Figure 4, showing a ~95% reduction in cell viability post 40 min of light on the Ant-PIm (10 μM, 24 h). By using Ant-PHEA under the same conditions (10 μM, 24 h), the cell death was only ~30% during 40 min of light. To achieve a higher effect of Ant-PHEA, some initial studies were performed using 50 μM and 100 μM (24 h) together with 30 min of light, resulting in a cell death of ~17% and 12%, respectively (data not shown). The decrease in cell survival during 3–5 min of blue light on Ant-PHEA incubated CHO-K1 cells (50 μM and 100 μM) was observed earlier in the bladder cancer cell line, AY27, after incubation by Ru-porphyrin [28] and in human colon cancer cells (WiDr) [29]. This phenomenon can partly be explained by the DNA repair mechanisms in cells, who work well at low blue light doses and were demonstrated in WiDr cells by 5-aminolevuline acid [29]. The 100% cell survival are results from control cells who followed the same experimental protocol, but without any chromophore, nor light. The cell viability as a % of treated culture dishes was calculated relative to the control cells. Furthermore, the dark toxicity effects of AntPIm alone (10 μM) resulted in a relatively low cell viability 62% (s.d. 7%; N = 4), but together with a short illumination time (1 min), the cell damage is partly repaired. For Ant-PHEA (10 μM), the viability in dark was found to be 87% (s.d. 9%; N = 2). These viability numbers correspond to dark toxicities of approx. 38% for Ant-PIm and 13% for Ant-PHEA.

**Figure 5:**
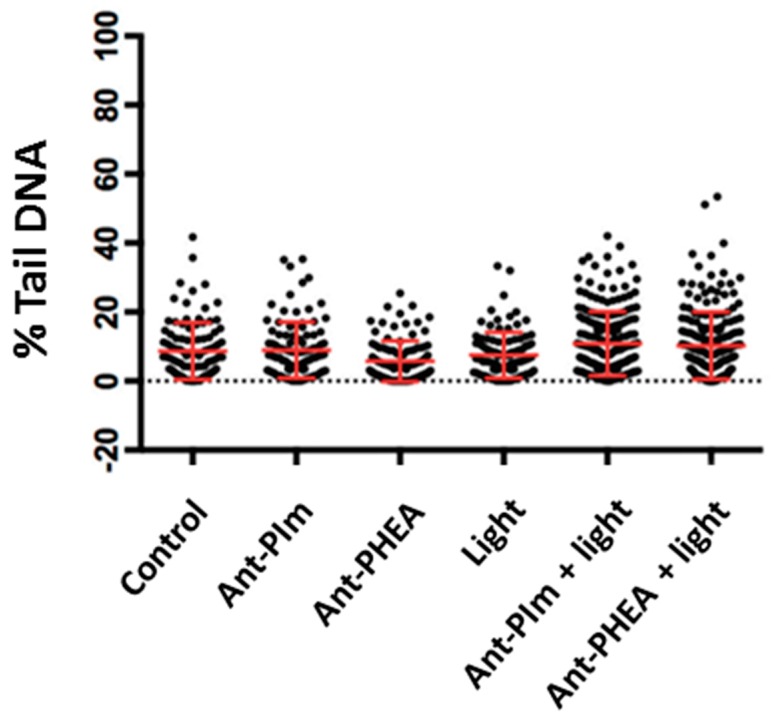
Comet analysis of the CHO-K1 hamster ovary cells presenting control cells (no treatment), the anthracenes alone (Ant-PIm, Ant-PHEA), light alone (30 s), and PDT-based anthracene (ANT-PIm + Light (30 s), Ant-PHEA + Light (30 s), both 2.5 µM, 24 h). The comet assay was performed immediately after light exposure, as described in the “Materials and Methods”. The data are one representative experiment out of three independent runs. Mean ± SD, *n* = 100.

In order to clarify the mechanisms of those dark- and light-induced toxicities and whether it relates to DNA alterations, further investigations were performed using comet assay [30]. The comet assays indicated only a marginal DNA damage upon Ant-PIm- or Ant-PHEA-mediated PDT immediately after 30 s of blue light exposure compared to the controls (Figure 5). A longer illumination period (3 min) was also performed but without a higher amount of %-tailed DNA (data not shown). This suggest that the PDT effect is not significantly associated with DNA damage, as would be expected in case of accumulation of the probe within the nuclei. This point was thus verified in the next series of experiments.

### 2.3. Localization of Ant-PIm and Ant-PHEA in Cells

The CHO-K1 cells stained with Ant-PIm or Ant-PHEA were co-stained together with Lysotracker red (far-red dye labelling lysosomes), DRAQ5 (DNA dye), or Mitotracker deep red (far-red dye labelling mitochondria), as outlined in the experimental section. 

**Figure 6:**
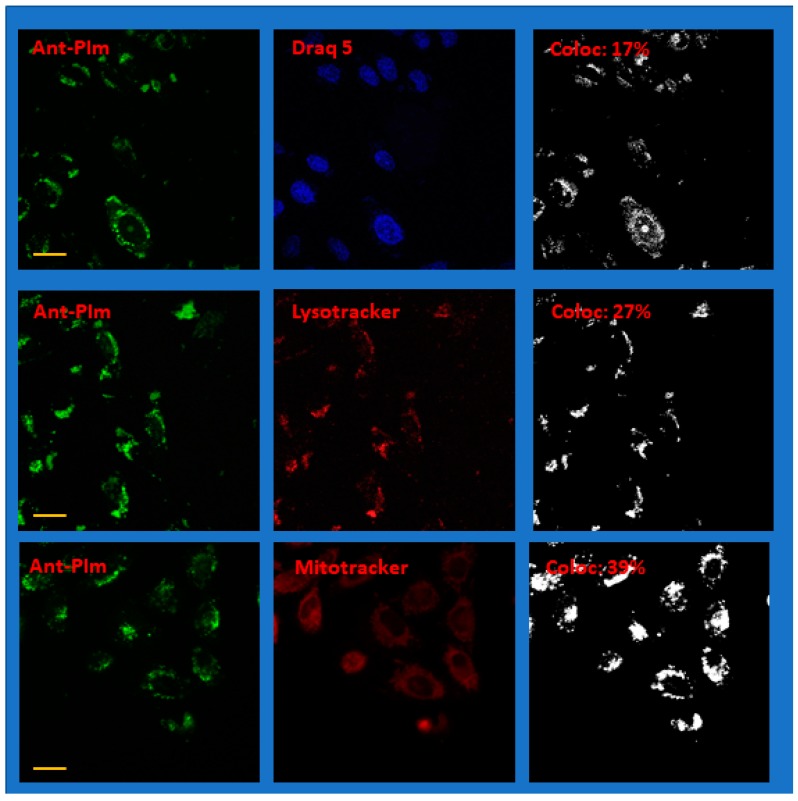
Representative confocal fluorescence microscope images of CHO-K1 hamster ovary cells after co-staining Draq 5, Lysotracker red, or Mitotracker deep red with Ant-PIm. The left panels are imaging the photo-sensitizer channel and the center panels the co-stained channel. The images in the right panels show the colocalization with the parameter for the whole image given as an inset (%). N.b. The colored images have been modified for clarity by adding brightness. The colocalization data are, by definition, binary and coded black and white. The yellow scale bar is 20 μm. For more details, see the text.

**Figure 7:**
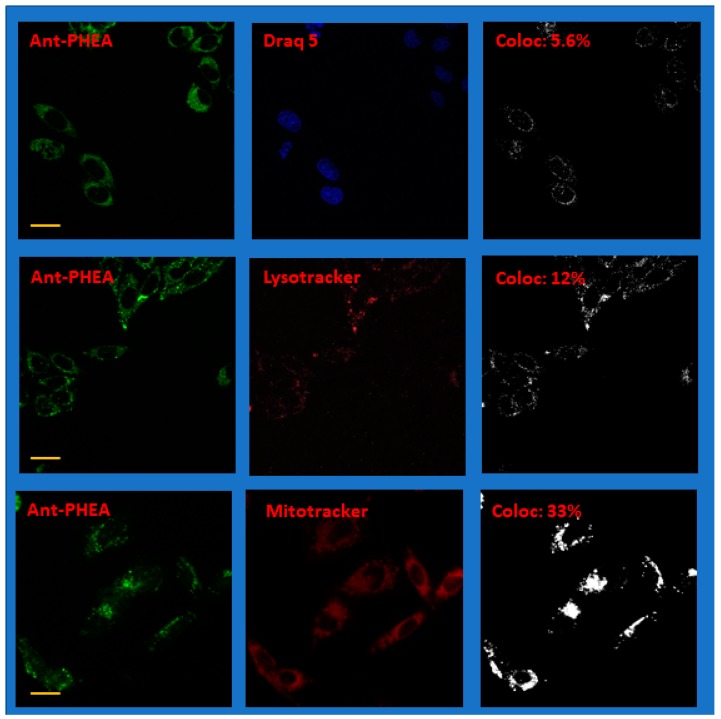
Representative confocal fluorescence microscope images of CHO-K1 hamster ovary cells after co-staining Draq 5, Lysotracker red, or Mitotracker deep red, with Ant-PHEA. The left panels are imaging the photo-sensitizer channel and the center panels the co-stained channel. The images in the right panels show the colocalization with the parameter for the whole image given as an inset (%). N.b. The colored images have been modified for clarity by adding brightness. The colocalization image is, by definition, binary and coded black and white. The yellow scale bar is 20 μm. For more details, see the text.

As judged from the absorption spectra in Figure 2, both Ant-PIm and Ant-PHEA (excitation maximum 489 nm and emission >505 nm) are readily excited with blue settings (435 nm), whereas both Lysotracker red and DRAQ5 are optimally selected by a red setting with excitation at 561 nm and Mitotracker deep red by a red setting using 633 nm as excitation wavelength. Care was taken to obtain images where each channel of the doubly-stained sample had intensity values (255 levels for each color) being safely below saturation. Representative images of doubly-stained cells using Draq 5, Lysotracker red, and Mitotracker deep red together with Ant-PIm and Ant-PHEA, are shown in Figure 6 and Figure 7, respectively, where each of the color channels are presented separately. 

More data of the same character as in Figure 6 and Figure 7 are shown in the Supplementary information, Figures S1–S6. Note that the images in the figures have been enhanced in brightness for clarity. 

In order to calculate the colocalization factor, each raw image of the separate channels containing the photosensitizer and the co-stain was first investigated to obtain an approximation of the background noise. Thus, a portion of each image (typically 25 × 25 pixels) containing no visible cells or other features was used to estimate a noise level. Typical intensity values were in the range 10–15 (depending on the image), and this noise level value ×2 was used to set a threshold when calculating the colocalization parameter as outlined in the methods section. The colocalization of each pixel is binary and represented for each image as the right panels of Figure 6 and Figure 7 as a black-and-white image. Accumulating double stained images from two separate occasions for each of the case, an average colocalization parameter was calculated, as summarized in Table 1, along with the standard deviation. 

## 3. Discussion

The two photo-sensitizers showed very similar absorption spectra but the emission of Ant-PIm shifted by approx. 15 nm in water or physiological serum. This is a direct consequence of the ionic nature of the polymer chain, which reinforces the polarity of the chromophore’s local environment, thereby affecting the excited state relaxation processes. Quite remarkably, the fluorescence quantum yield is similar in both cases. The value 0.3 is lower than initially reported for Ant-PHEA [11], presumably because of the shorter length of the polymer scaffold (5 vs. 11 repetitive units), which provides a less efficient shielding against chromophore–water interactions. This value, obtained using water as solvent, remains high in that range of wavelength, making both compounds well-suited for bioimaging applications. In spite of the shortness of the polymer chain, solubility remains remarkably high in a biological medium; both chromophores can be readily dissolved in water or saline buffer at concentrations exceeding 100 g/L. This value ensures the possible use of the chromophore for virtually all applications requiring physiological solubility.

Concerning the singlet oxygen luminescence, it could not be detected for either Ant-PIm or Ant-PHEA. Instead, their transients at 1275 nm show a very short response that might be due to some residual emission from the tail of their fluorescence. One might also speculate upon a weak emission from some short-lived triplet state that does not transfer energy to or become quenched by the oxygen in the solvent. On the other hand, the reference sample of Erythrosin B showed typical luminescence with decay in accordance with the previously reported 70 μs for singlet oxygen decay in D_2_O [26,27]. Conclusively, the transients of the sensitizers Ant-PIm and Ant-PHEA do not reflect singlet oxygen. This feature nicely matches our initial hypothesis and points onto a PDT effect that does not rely on a classical type II mechanism. 

Turning to the cell viability measurements, as summarized in Figure 4, it was clear that light alone did not induce significant cell death. By comparison, the introduction of the Ant-PIm and Ant-PHEA sensitizers resulted in significant cell death. In that regard, Ant-PHEA has an evidently smaller dark toxicity (13% cell death) compared to Ant-PIm (38% cell death). Such toxicity of polycationic chromophores was recently reported by Chennoufi et al., and it was noticed that it was accompanied with subsequent localization of the chromophore within the cell nucleus upon cell death [24], similar to the case with Ant-PIm. In fact, dead cells with Ant-PIm in the nucleus was observed also after our experiments (data not shown). Concerning the light induced toxicity, the initial drop in the Ant-PHEA curve during the first 1–3 min of light exposure (Figure 4) is often observed in PDT-cell survival studies [29] and, as reported previously, also on a well-studied bladder cancer cell line, AY27, after TPCS_2a_-based PDT [31]. The dark toxicity (0 min of light) recorded for Ant-PIm was initially high (38%) but, together with the blue-light-induced cell death of 0–3 min, the repairing mechanisms work for this short period. The combination of the photosensitizer Ant-PIm and longer illumination periods (more than 3 min) resulted in a cell death that could not be repaired, and the cell survival dropped to almost zero after 20 min of illumination. After the partial recovery, there was no further substantial cell death indicating an overall low PDT efficiency (30–40%) of the Ant-PHEA molecules during illumination of 40 min, which agrees with published data on human colon adenocarcinoma cells (WiDr) [29]. The marginal DNA damage indicated by the comet assay (Figure 5) indicates that although DNA-targeted [32] destruction could participate in the cell apoptotic process, it is not the main mechanism responsible for the observed photo induced cell toxicity immediately after light treatment. Thus, PDT might have stimulated some signal pathways leading to cell apoptotic death.

The differences can be put in line with the drastically different localization observed for both compounds. From the images and colocalization experiments (Figure 6 and Figure 7, Table 1), it is apparent that Ant-PHEA and Ant-PIm markedly differ in their accumulation within different cell compartments. Whereas Ant-PHEA provides a rather uniform staining of the cytosol and a few spots of the nucleus, Ant-PIm exhibits a stronger affinity towards lysosomal compartments as well as mitochondria. Moreover, a significant higher fraction (13%) of Ant-PIm is found within the cell nucleus as compared to Ant-PHEA (5%). 

Thus, in cellular domains of metabolic importance, such as mitochondria, lysosome, and (marginally) the cell nucleus, Ant-PHEA is far less effective than that of Ant-PIm. Numerous studies have pointed out the influence of mitochondrial oxidative stress on cell apoptosis processes1 [6,33,34]. Therefore, it can be anticipated that Ant-PIm, because of its localization, is more prone than its non-cationic counterpart to inducing damage on the cell machinery upon photo-irradiation, to an extent that strongly compromises cell survival, which verifies from the cell survival upon light exposure data; unfortunately, this mitochondrial localization also translates into a somewhat higher dark cytotoxicity. The immediate decrease in cell survival during 3–5 min of blue light exposure, with the subsequent increase in cell survival, has been observed on rat bladder cancer cells (AY27) after incubation by Ru-porphyrin [28] and in human colon cancer cells (WiDr) by 5-aminolevuline acid (ALA)-based PDT. This phenomenon can partly be explained by the DNA repair mechanisms in cells, which work well at low blue light doses and are well demonstrated by ALA-based PDT [29]. The drop of cell survival in CHO-K1 cells post 24 h incubation increased by higher concentrations (50 μM or 100 μM) in combination with blue light.

Conclusively, the cell localization and toxicities of two related anthracene chromophores was compared. The sensitizers differ only by the nature of the appended polymer chains, more specifically by the presence or absence of cationic charges. None of the chromophores generated singlet oxygen upon photo-illumination, even though a significant cell death was observed with Ant-PIm. This could indicate a possible type I PDT mechanism. Type I PDT is covering a broad range of photoinduced redox mechanisms and reactive oxygen and nitrogen species, and it is beyond the scope of the present study to elucidate its detailed mechanisms. The question of generated reactive species is left for future studies, for instance, by means of EPR spectroscopy. As a somewhat contrasted conclusion, while Ant-PIm showed, owing to its improved penetration in important sub compartments of the cell machinery, considerably larger PDT efficiency than its neutral counterpart Ant-PHEA, it also comes with larger dark toxicity. Indirect DNA damage, as quantified by the comet assay, partly showed a small decrease in the repair kinetics immediately after anthracene-based PDT, although the extent of those damages seemed too small to compromise cell survival to such an extent as monitored. Thus, the exact mechanism at the origin of cell toxicity is not entirely understood. The latter issue must be addressed on in future work by detecting damage after longer post incubation periods for eventually documenting some apoptotic cell deaths.

## 4. Materials and Methods 

### 4.1. Chemicals

RPMI-1640 medium, l-glutamine, fetal bovine serum (FBS), sodium pyruvate, nonessential amino acids, trypsin, and phosphate buffered saline (PBS) were obtained from Gibco BRL, Life Technologies (Inchinnan, UK). Gentamicin sulphate was purchased from Schering Corp (Kenilworth, NJ, USA) and absolute ethanol from Arcus A/S (Oslo, Norway). The MTT solution 3-(4, 5 dimethylthiazol-2-yl)-2,5-diphenyltetrazoliumbromide) was purchased from Sigma-Aldrich (St. Louis, MO, USA). The fluorochrome DRAQ5 were ordered from Invitrogen, Eugene, OR, USA, Lyso Tracker Red from eBioscience, Inc., San Diego, CA and Mito Tracker Deep Red from Molecular Probes, Inc., Invitrogen, Eugene, OR, USA. Chromophores from ENS-Lyon, (Cyrille). Other chemicals were of the highest quality commercially available.

### 4.2. Chromophores

Methodologies for the synthesis of Ant-PHEA [11] and Ant-PIm [21] have been reported elsewhere. Their structure is depicted in Figure 1. Polymer chain length was adjusted, owing to the controlled polymerization process, so that *n* = 5, which corresponds to the minimal length that ensures water solubility at biologically relevant concentration, and which hopefully should be short enough to maintain a good hydrophilic–lipophilic balance and therefore enhance cell permeation (vide supra) [35]. The chromophores were dissolved in dH_2_O preparing a stock solution (100 µM) before further dilution in a growth medium.

### 4.3. Cell Culture

The hamster ovary cell line CHO-K1 was cultured in Corning/Sarstedt, 60 mm × 15 mm dishes, Nunc Denmark, and grown in F-Nutrient Mixture, containing 10% *v*/*v* fetal bovine serum (FBS), l-glutamine (80 mg/L), penicillin (100 U/mL), streptomycin (100 U/mL), and fungizone (0.25 mg/mL). The cell cultures were maintained at 37 °C in an incubator in an atmosphere of 5% CO_2_, 95% air, and were subcultured approximately twice a week.

### 4.4. Optical Spectroscopy

UV-visible spectra were recorded on a Jasco^®^ V-670 spectrophotometer in diluted physiological serum solutions (NaCl aq. 0.9 wt %). Luminescence spectra were measured using a Horiba-Jobin-Yvon Fluorolog-3^®^ spectrofluorometer, equipped with a three-slit double-grating excitation and emission monochromator with dispersions of 1200 grooves/mm and an R928 detector. The spectra were reference corrected for both the excitation source light intensity variation (lamp and grating) and the emission spectral response (detector and grating). Fluorescence quantum yields Q were measured in diluted water solution with an optical density lower than 0.1 using the Equation (1):

(1) $$\frac{Q_{x}}{Q_{r}}=\left[\frac{A_{r}\left(\lambda \right)}{A_{x}\left(\lambda \right)}\right]\left[\frac{n_{x}^{2}}{n_{r}^{2}}\right]\left[\frac{D_{x}}{D_{r}}\right],$$

where *A* is the absorbance at the excitation wavelength (λ), *n* is the refractive index, and *D* is the integrated luminescence intensity. “*r*” and “*x*” stand for reference and sample. Here, reference was fluorescein in basic (pH = 13) aqueous solution (*Q_r_* = 0.91) [20]. 

### 4.5. MTT Assay Cell Survival after Blue Light PDT

The CHO-K1 cells were seeded in Petri dishes (ϕ = 6 cm, Nunc Denmark) at a density of 0.25–0.35 × 10^6^ cells per dish (one day prior to PDT experiment) using a regular cultivation medium. A stock solution of the chromophores (Figure 1) was prepared in absolute ethanol or distilled H_2_O prior to further dissolving in a growth medium. After washing (PBS), the ordinary medium containing the chromophores (10 µM, 18 h, 37 °C, 5% CO_2_) were added in the dark, and the dishes washed in PBS before being exposed to blue light (435 nm, 13 mW/cm^2^) and incubated overnight (37 °C, 5% CO_2_). Cells containing only chromophores were considered for “dark toxicity” assessments and samples without any treatment (nor light or photosensitizer) were used as controls. After a post incubation period (24 h, 37 °C, 5% CO_2_) from illumination, the MTT proliferation assay was performed [36] as follows: the culture medium was removed and the cells were incubated in MTT-solution (0.5 mg/mL, 1 h, 37 °C, 5% CO_2_ (Sigma-Aldrich, St. Louis, MO, USA) before replacing by isopropanol (2 mL) and placing on a plate shaker (30 min, 80 rpm). The important and modified step, as described by Gederaas et al. [31], is the removal of dead cells by centrifuging (5 min, 1500 rpm). The absorbance (595 nm) was measured using a Shimadzu UV-1700 spectrophotometer. The obtained data were processed and compared to the untreated cells. The cells were illuminated with blue light from below by using the LumiSource^®^ from PCI Biotech AS, Oslo, Norway, consisting of 4 Osram tubes (emission maximum = 435 nm, 13 mW/cm^2^). The light was detected near the bottom of the cell dishes. Both light-sensitive solutions and cells were covered with aluminum foil during the whole experiment.

### 4.6. Comet Assay

UV cells were seeded out in six-well cell plates (1 × 10^4^ cells/well, CytoOne) and were incubated overnight (37 °C, 5% CO_2_) before anthracene incubation (2.5 µM, 24 h, 5% CO_2_). Immediately after light exposure (30 s or 3 min) the DNA damage level was further evaluated using a standard protocol of single cell gel electrophoresis [30,37]. After being suspended in low melting agarose gel (1%), the cell suspension (75 µL) was put on a pre-coated microscope slide (in duplicates), which was immediately cooled down on ice. Embedded cells were lysed in fresh cold lysis buffer [30] overnight (4 °C) and then electrophoresed in cold alkaline buffer (pH 13.3). DNA fragments stained with ethidium bromide were visualized in an inverted fluorescence microscope (Zeiss Axiovert 200M, Zeiss, Oberkochen, Germany) with an RHOD filter (535–585 nm) connected to a Sony XCD-X700 camera. The imaging software Kinetic Comet 5.5 (Andor Technology, Belfast, UK) was used to pick and evaluate 100 comets randomly.

### 4.7. Singlet Oxygen Detection

UV for photophysical measurements standard 1 cm UV quartz cuvettes (Hellma) were employed, having Teflon caps that allow flushing with Argon gas to remove oxygen from the solvent. MeOH and D_2_O were used as solvents (Merck Life Science AS, Oslo, Norway). As a reference standard, Erythrosin B (purity > 95% purity; Merck) was used, which is known to give a high yield (>60%) of singlet oxygen in both water and ethanol upon irradiation below 530 nm [25]. The singlet oxygen formation was demonstrated by recording the transient singlet oxygen luminescence (at 1275 nm) of solutions with the sample in a standard 90° configuration (excitation and emission path). A tunable OPO laser, NT 342A-SH-10-WW (Ekspla, Lithuania) was used for excitation in the range 425–460 nm. For the detection a PMT (R5509, Hamamatsu), an interference filter with maximum transmission at 1272.5 nm and a long pass filter transmitting above 780 nm were used. An Infiniium BDSU Oscilloscope (Keysight technologies, Santa Rosa, CA, USA) was used to sample and collect data. Time-gated electronics were used to control the time between laser excitation and the recording of the luminescence transient. The transients were background corrected by subtracting with the signal of the pure solvents. In order to remove oxygen in the solvent, the cuvette was purged with Argon for 10 min. A similar procedure for confirming the singlet oxygen production of Platinum and Ruthenium complexes for PDT was recently presented in Bogoeva et al. [28] and Chatzideri et al. [38].

### 4.8. Confocal Microscopy and Colocalization

UV CHO-K1 cells were seeded (0.3 × 10^6^ cells per dish) on glass-bottom dishes (ϕ = 3.5 cm) from MatTek corporation, Ashland, MA, USA, and were incubated (24 h, 37 °C) with/without Ant-PIm/Ant-PHEA (10 µM) in combination with/without Lysotracker red (50 nM, 1 mL, 1 h, 37 °C, 5% CO_2_), with/without DRAQ5 (1 µM, 1 mL, 15 min 37 °C, 5% CO_2_), or with/without Mitotracker deep red (1 µM, 30 min, 37 °C, 5% CO_2_). The cells were imaged with confocal laser scanning microscopy (Zeiss LSM 5 DUO, Zeiss 510 META, Zeiss LSM 5 Live, Germany), post 24 h treatment, using a plan apochromatic 63 × oil immersion objective with numerical aperture 1.4. Ant-PIm and Ant-PHEA were both excited with a 489 nm laser and emission detected using an LP505 filter. Lysotracker red and Draq 5 were excited using a 561 nm laser and were detected with BP575-614 and LP650 filters, respectively. Mitotracker deep red was excited using 633 nm (He-Ne laser) and detected with LP 650 filter. The excitation period during the images was about 5 s, with little change for the re-localization of the compound, and the imaging was performed twice in 2–3 biological replicates, recorded at two different occasions. In our experience, the imaging of living cells is safer than confocal studies on fixed cells.

All emissions where detected through a 1 Airy Unit pinhole. The laser power, detector gain, and offset were chosen to minimize auto-fluorescence in the control samples where no photosensitizer was added. For further processing, 512 by 512-pixel images with an 8 bit dynamic range was recorded. The procedure for the colocalization assessment of CHO-K1 cells using the chromophores Ant-PIm and Ant-PHEA, together with the markers Lysotracker red, Draq 5, and Mitotracker deep red, was based on the algorithms and software analysis developed in-house using the Matlab (Mathworks) mathematics software package. Briefly, the brightness and dye concentration of each image can differ, so a normalized colocalization parameter was defined, as shown in Equation (2):

(2) $$C_{f}\left(\%\right)=100\times \frac{N_{cp}}{\left(\left(N_{1}+N_{2}\right)-N_{cp}\right)},$$

where *N_cp_* is the number of pixels in the colocalized image, and *N*_1_ and *N*_2_ are the number of pixels over the threshold for the individual images to be compared. This formula gives the value 1 at the limit when all pixels are colocalized and goes to zero when no pixels are colocalized, as might be expected. This allows us to make a statistical assessment of the colocalization in different cells and compare different cell cultures. Regions of low signal (no visible cells or other features) were used to estimate the noise level of each wavelength channel, and this was used to set a threshold value for the colocalization of each pixel, i.e., pixels with values under the threshold were not used.

## Figures and Tables

**Table 1:** Calculated colocalization factor C_f_ between Ant-PIm and Ant-PHEA with the commercial dyes Draq 5, Lysotracker red, and Mitotracker deep red. The calculated standard deviation is based on samples recorded at two different occasions from independently stained wells.

Chromophore	Stain	C_f_ (%)	SD (%), N Samples
Ant-PIm	DRAQ 5	13	4.6 (N = 6)
	Lysotracker	26	3.9 (N = 6)
	Mitotracker Deep Red	29	6.6 (N = 10)
Ant-PHEA	DRAQ 5	5.0	1.6 (N = 6)
	Lysotracker	7.9	3.5 (N = 6)
	Mitotracker Deep Red	25	9.5 (N = 4)

## Supplementary Materials

The following are available online. Figures S1–S6: Supplementary confocal images.
