# Supplementary material for: Influence of Polymer Charge on the Localization and Dark- and Photo-Induced Toxicity of a Potential Type I Photosensitizer in Cancer Cell Models
Source: Molecules. 2020 Mar 3;25(5):1127. doi: 10.3390/molecules25051127 (PMC7179247; doi:10.3390/molecules25051127)
Supplement: Supplementary file 1 [file molecules-25-01127-s001.pdf]

Supplementary information

Influence of Polymer Charge on the Localization and Dark- and Photo-Induced Toxicity of a Potential Type I Photosensitizer in Cancer Cell Models

Mikael Lindgren [1,*], Odrun A. Gederaas [1,2], Monica Siksjø [1], Tom A. Hansen [1], Lena Chen [3], Bastien Mettra [3], Chantal Andraud [3] and Cyrille Monnereau [3]

[1] Department of Physics, Faculty of Natural Sciences, Norwegian University of Science and Technology, Gløshaugen, NO-7491 Trondheim, Norway; odrun.gederaas@ntnu.no (O.A.G.); m.siksjo@gmail.com (M.S.); tomhans@viken.no (T.A.H.)

[2] Department of Clinical and Molecular Medicine, Faculty of Medicine and Health Sciences, Norwegian University of Science and Technology, Laboratoriesentret 5, NO-7491 Trondheim, Norway

[3] Laboratoire de Chimie, CNRS UMR 5182, ENS de Lyon, Université Lyon 1, F-69342 Lyon, France; chenlena93@gmail.com (L.C.); bastien.mettra@univ-lyon1.fr (B.M.); chantal.andraud@ens-lyon.fr (C.A.); cyrille.monnereau@ens-lyon.fr (C.M.)

* Correspondence: mikael.lindgren@ntnu.no; Tel.: +47-414-66-510

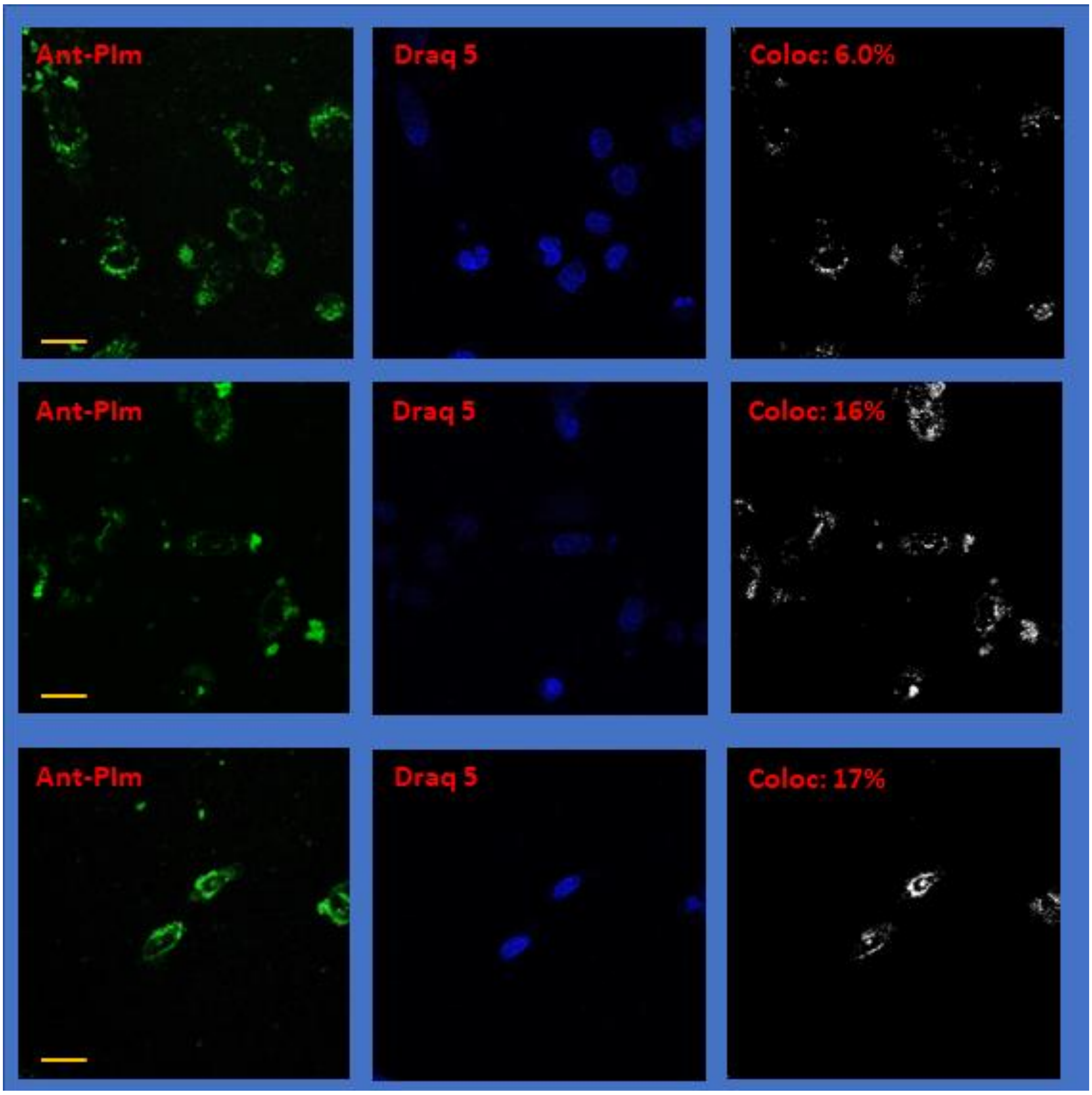

Figure S1. Three examples of confocal images of CHO-K1 cells with Draq 5co-stained with Ant-PIm. Left panels are imaging the photo-sensitizer channel and center panels the co-stained channel. The images in the right panels shows the colocalization with the parameter for the whole image given as an inset (%). N.b. the colored images have been modified for clarity by adding brightness. The colocalization data is by definition binary, coded black and white. Yellow scale bar is 20 μm. For more details see the text.

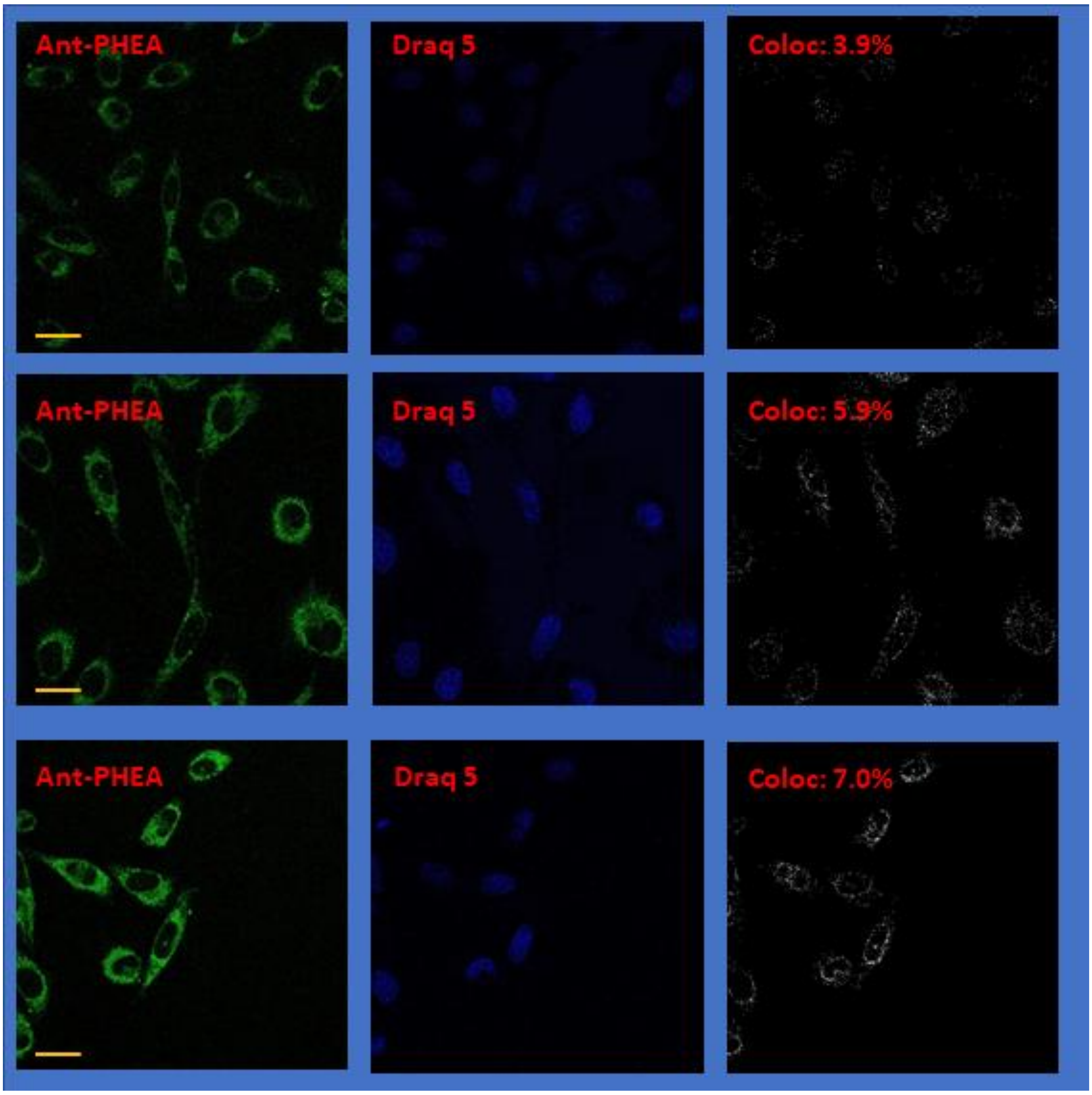

Figure S2. Three examples of confocal images of CHO-K1 cells with Draq 5 co-stained with Ant-PHEA. Left panels are imaging the photo-sensitizer channel and center panels the co-stained channel. The images in the right panels shows the colocalization with the parameter for the whole image given as an inset (%). N.b. the colored images have been modified for clarity by adding brightness. The colocalization data is by definition binary, coded black and white. Yellow scale bar is 20 μm. For more details see the text.

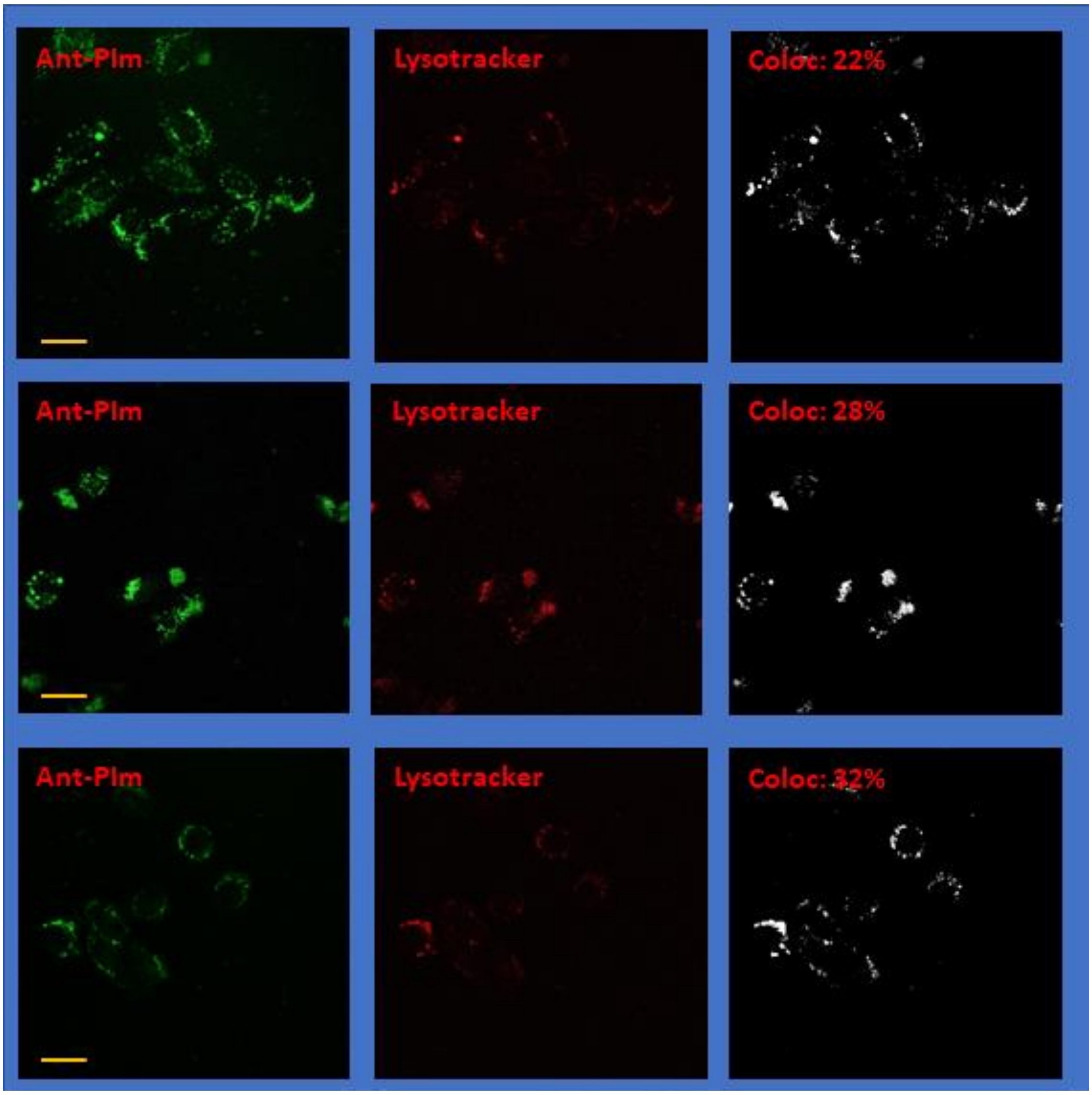

Figure S3. Three examples of confocal images of CHO-K1 cells with Lysotracker red co-stained with Ant-PIm. Left panels are imaging the photo-sensitizer channel and center panels the co-stained channel. The images in the right panels shows the colocalization with the parameter for the whole image given as an inset (%). N.b. the colored images have been modified for clarity by adding brightness. The colocalization data is by definition binary, coded black and white. Yellow scale bar is 20 μm. For more details see the text.

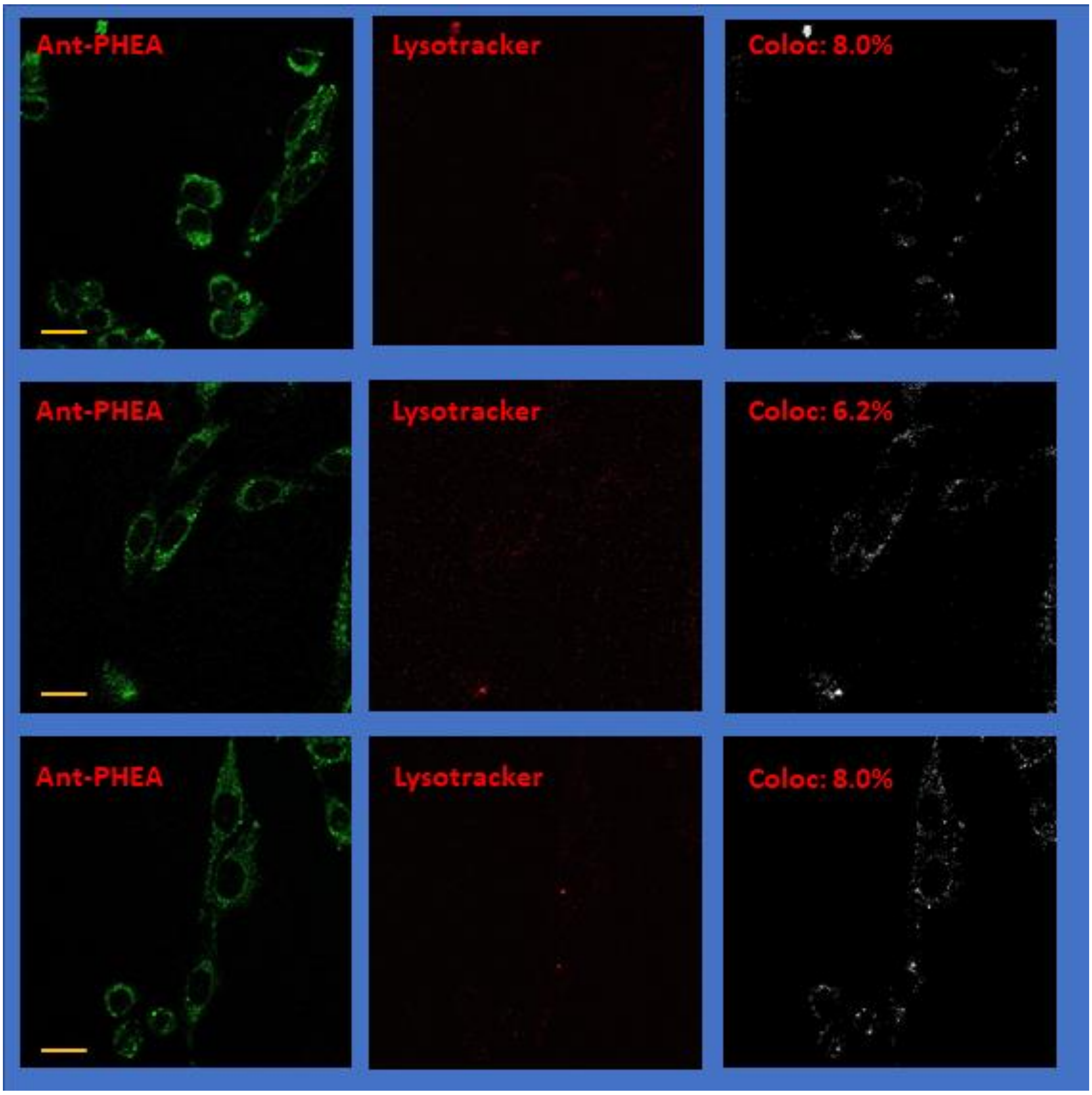

Figure S4 Three examples of confocal images of CHO-K1 cells with Lysotracker red co-stained with Ant-PHEA. Left panels are imaging the photo-sensitizer channel and center panels the co-stained channel. The images in the right panels shows the colocalization with the parameter for the whole image given as an inset (%). N.b. the colored images have been modified for clarity by adding brightness. The colocalization data is by definition binary, coded black and white. Yellow scale bar is 20 µm. For more details see the text.

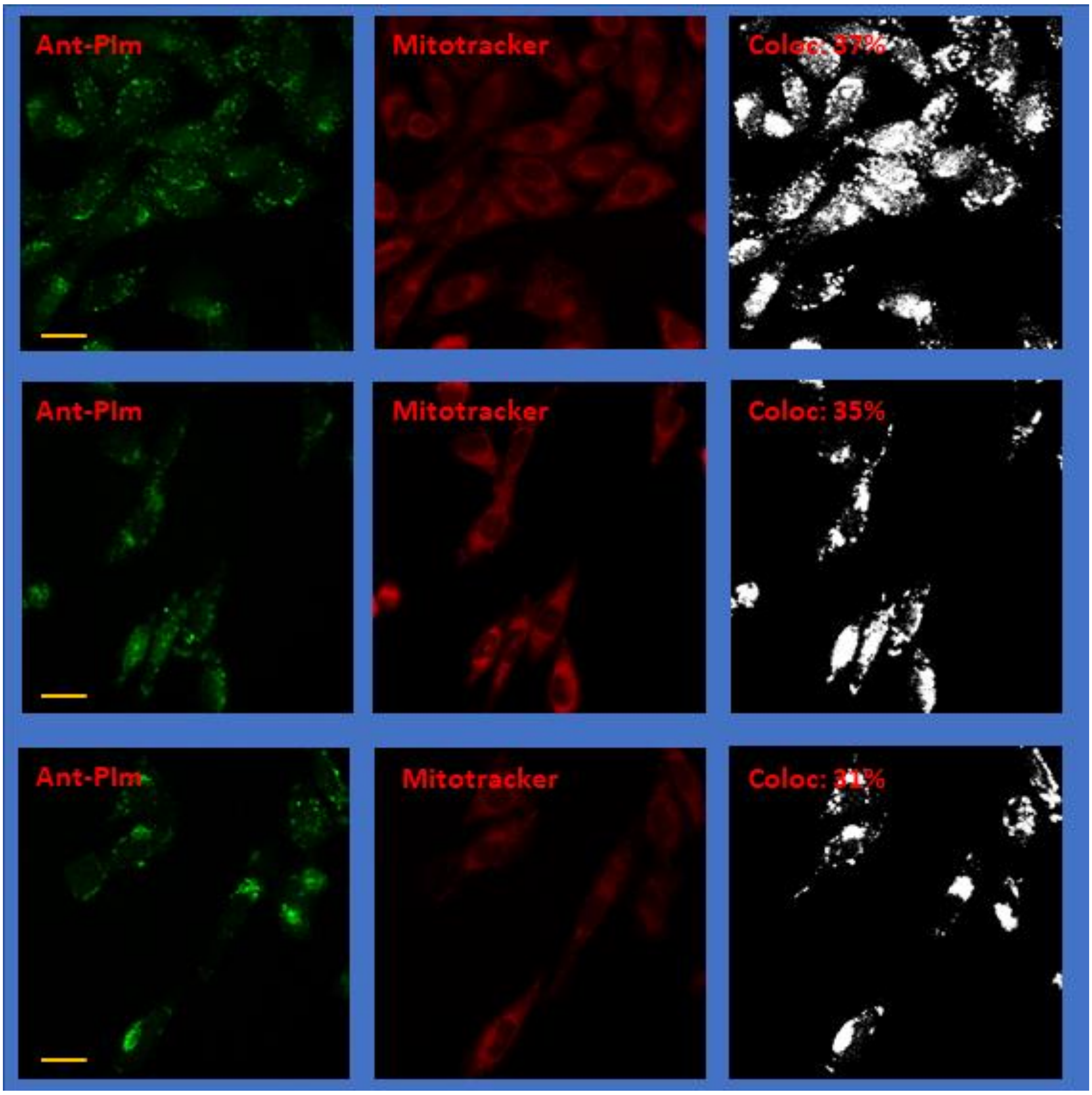

Figure S5. Three examples of confocal images of CHO-K1 cells with Mitotracker deep red co-stained with Ant-PIm. Left panels are imaging the photo-sensitizer channel and center panels the co-stained channel. The images in the right panels shows the colocalization with the parameter for the whole image given as an inset (%). N.b. the colored images have been modified for clarity by adding brightness. The colocalization data is by definition binary, coded black and white. Yellow scale bar is 20 μm. For more details see the text.

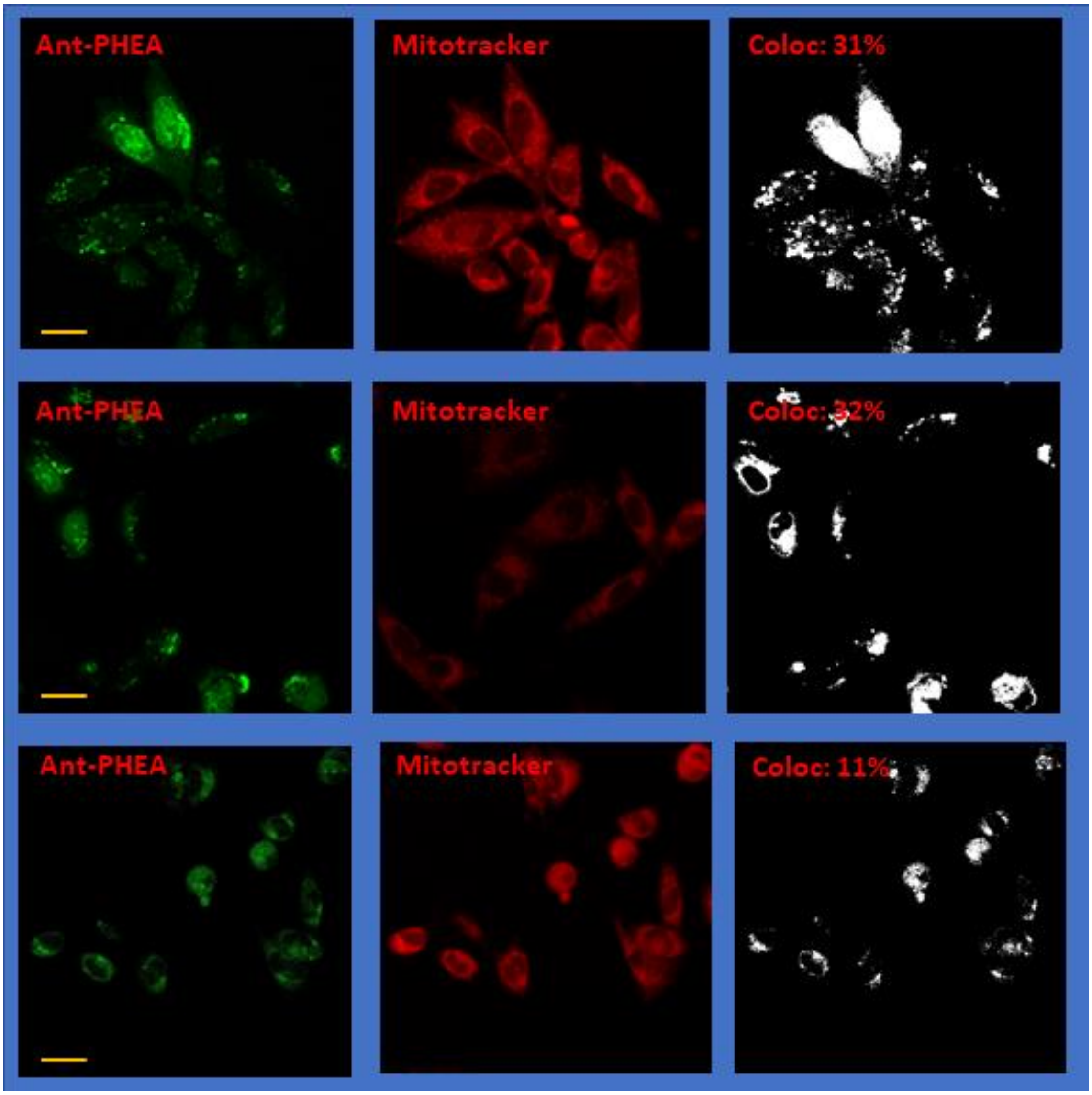

Figure S6. Three examples of confocal images of CHO-K1 cells with Mitotracker deep red co-stained with Ant-PHEA. Left panels are imaging the photo-sensitizer channel and center panels the co-stained channel. The images in the right panels shows the colocalization with the parameter for the whole image given as an inset (%). N.b. the colored images have been modified for clarity by adding brightness. The colocalization data is by definition binary, coded black and white. Yellow scale bar is 20 μm. For more details see the text.
